# Assessing bacterial diversity in a seawater-processing wastewater treatment plant by 454-pyrosequencing of the 16S rRNA and amoA genes

**DOI:** 10.1111/1751-7915.12052

**Published:** 2013-04-10

**Authors:** Olga Sánchez, Isabel Ferrera, Jose M González, Jordi Mas

**Affiliations:** 1 Departament de Genètica i Microbiologia, Universitat Autònoma de Barcelona08193, Bellaterra, Spain; 2 Department of Microbiology, University of La LagunaES-38206, La Laguna, Tenerife, Spain

## Abstract

The bacterial community composition of activated sludge from a wastewater treatment plant (Almería, Spain) with the particularity of using seawater was investigated by applying 454-pyrosequencing. The results showed that *Deinococcus*-*Thermus*, *Proteobacteria*, *Chloroflexi* and *Bacteroidetes* were the most abundant retrieved sequences, while other groups, such as *Actinobacteria*, *Chlorobi*, *Deferribacteres*, *Firmicutes*, *Planctomycetes*, *Spirochaetes* and *Verrumicrobia* were reported at lower proportions. Rarefaction analysis showed that very likely the diversity is higher than what could be described despite most of the unknown microorganisms probably correspond to rare diversity. Furthermore, the majority of taxa could not be classified at the genus level and likely represent novel members of these groups. Additionally, the nitrifiers in the sludge were characterized by pyrosequencing the *amoA* gene. In contrast, the nitrifying bacterial community, dominated by the genera *Nitrosomonas*, showed a low diversity and rarefaction curves exhibited saturation. These results suggest that only a few populations of low abundant but specialized bacteria are responsible for removal of ammonia in these saline wastewater systems.

## Introduction

Activated sludge constitutes a crucial tool in the biodegradation of organic materials, transformation of toxic compounds into harmless products and nutrient removal in wastewater treatment plants (WWTPs). It contains a highly complex mixture of microbial populations whose composition has been intensively studied in the past decades. By applying culture-dependent methods many species have been isolated from activated sludge (Dias and Bhat, 1964; Prakasam and Dondero, 1967; Benedict and Carlson, 1971). However, a great majority cannot be obtained by conventional techniques (Wagner *et al*., 1993) and, consequently, current molecular techniques such as sequence analysis of 16S rRNA gene clone libraries (Snaidr *et al*., 2011), fingerprinting methods such as denaturing gradient gel electrophoresis (DGGE; Boon *et al*., 2002), thermal gradient gel electrophoresis (TGGE; Eichner *et al*., 1964) and terminal restriction fragment length polymorphism (Saikaly *et al*., 2011) along with fluorescence *in situ* hybridization (FISH) have been employed in wastewater microbiology to analyse and compare the microbial structure of activated sludge. Recently, PCR-based 454 pyrosequencing has been applied to investigate the microbial populations of activated sludge in different WWTPs as well as in full-scale bioreactors (Sanapareddy *et al*., 2009; Kwon *et al*., 2010; Kim *et al*., 2011; Ye *et al*., 2002; Zhang *et al*., 2011a; b), greatly expanding our knowledge on activated sludge biodiversity.

An important process in WWTPs is nitrification, in which ammonium is removed by converting it first into nitrite and then to nitrate. Different bacterial species involved in this process have been characterized by means of clone library analysis in addition to FISH (Juretschko *et al*., 1998; Purkhold *et al*., 2000; Daims *et al*., 2001; Zhang *et al*., 2011b). Several ammonia-oxidizing and nitrite-oxidizing bacterial populations belonging to the phylum *Nitrospira* and to *Beta*-and *Gammaproteobacteria* have been identified as key members in this process, such as the genera *Nitrosomonas, Nitrobacter, Nitrospira* and *Nitrosococcus* (Wagner *et al*., 2002; Zhang *et al*., 2011b). Nevertheless, most studies of microbial diversity in WWTPs refer to freshwater plants, either domestic or industrial, and yet very little is known about plants that utilize seawater for their operation, mainly because there are still very few of these running in the world. Their utilization responds to the deficiency in hydric resources prevailing in their locations and their use will probably increase in the near future due to water shortage associated to global warming as many areas are experiencing today (Barnett *et al*., 2005). As a consequence, knowledge of the microbial diversity becomes crucial to identify the key players in these systems.

In a recent survey (Sánchez *et al*., 2011), the prokaryotic diversity of a seawater-utilizing WWTP from a pharmaceutical industry located in the south of Spain was characterized using a polyphasic approach by means of three molecular tools that targeted the 16S rRNA gene, i.e. DGGE, clone libraries and FISH. The results showed that the composition of the bacterial community differed substantially from other WWTP previously reported, since *Betaproteobacteria* did not seem to be the predominant group; in contrast, other classes of *Proteobacteria*, such as *Alpha-* and *Gammaproteobacteria*, as well as members of *Bacteroidetes* and *Deinoccocus*-*Thermus* contributed in higher proportions. Besides, utilization of specific primers for amplification of the *amoA* (ammonia monooxygenase subunit A) gene confirmed the presence of nitrifiers corresponding to the *Beta*-subclass of *Proteobacteria*, although they were not identified in this study.

In the present article, we further investigated the diversity of this system by applying 454-pyrosequencing, a much more powerful molecular technique, which provides thousands of sequence reads. We analysed the bacterial assemblage by targeting the 16S rRNA gene and increased our knowledge on its diversity by one order of magnitude. Additionally, we characterized the nitrifying members of this sludge by pyrosequencing the *amoA* gene. As far was we know, this is the first study that analyses the *amoA* gene diversity in an activated sludge of a WWTPs with the particularity to utilize seawater.

## Results and discussion

We investigated the bacterial community structure and identified the nitrifying members from the activated sludge of a seawater-utilizing WWTP located in Almeria (South-east Spain). The plant treats wastewater from a pharmaceutical industry. The mean influent flow of the plant is 300 m^3^ h^−1^ and has a treatment volume of 32 000 m^3^. Nitrogen and chemical oxygen demand sludge loads were about 150–170 kg h^−1^ and 900–1000 kg h^−1^ respectively. DNA was extracted from samples of aerated mixed activated sludge collected in 2 consecutive years (2007 and 2008).

### Diversity of bacterial communities in activated sludge

**Figure 1:**
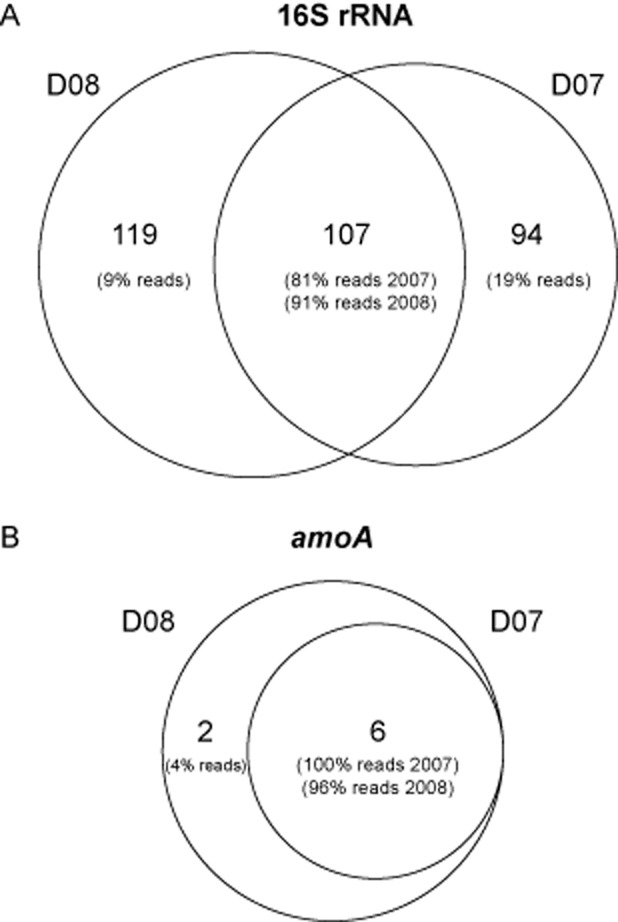
Venn diagrams of shared OTUs between the two samples (D07 and D08) for (A) 16S rRNA gene and (B) *amoA* gene. The number of reads that the OTUs represent is indicated in brackets.

After a rigorous quality control (see *Experimental procedures* and Table S1) a total of 16 176 16S rRNA tag sequences of sufficient quality were analysed (8010 sequences corresponding to year 2007 and 8166 sequences to year 2008) and grouped into operational taxonomic units using uclust at 3% cut-off level. The clustering resulted in a total of 320 different OTUs from which 107 were shared between samples (33.3%) as shown in the Venn diagram (Fig. 1A). The number of OTUs in 2007 was 201 and in 2008 was 226. Although the proportion of shared OTUs is rather low, the unique diversity in each sample corresponded mainly to rare OTUs (relative abundance below 1%). In the case of year 2007, the unique clones to that sample represented a 19% of the total reads, from which only three OTUs were above 1%. For the 2008 sample, the unique clones represented a 9% of total sequences and only two OTUs presented an abundance above 1%. These results are in agreement with previous observations in which DGGE analysis from both samples showed virtually the same pattern for universal primers amplifying Bacteria, suggesting that activated sludge was at a steady state at least for the most abundant phylotypes (Sánchez *et al*., 2011).

**Figure 2:**
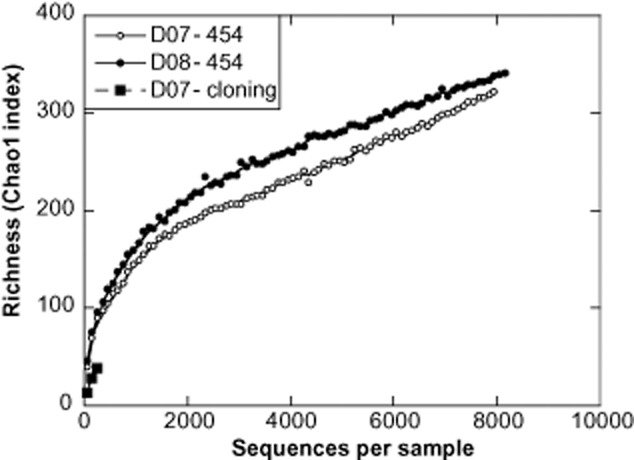
Rarefaction curves of 16S rRNA OTUs defined by 3% sequence variations in the activated sludge. Curves refer to the pyrosequence analyses of the 2 consecutive years (D07 and D08-454) compared with the clone library analysis of the 2007 sludge (D07 – cloning).

Richness was computed by the Chao1 estimator and analysis by rarefaction showed that the diversity in the two samples was within the same range, although slightly higher in 2008. However, we found that this depth of sequencing was not sufficient to saturate the curve and therefore, the actual diversity is likely much higher (Fig. 2). Nevertheless, if compared with the rarefaction curve from a clone library performed from the 2007 activated sludge sample, we observe that, by applying pyrosequencing we increased our knowledge on the diversity present by one order of magnitude. Rank-abundance curves (Fig. S1A) show that there were only a few abundant phylotypes and a long tail of rare taxa, therefore, most of the unknown diversity probably corresponds to rare diversity (Pedrós-Alió, 2007).

RDP Classifier was used to assign the representative OTU sequences into different phylogenetic bacterial taxa. Figure S2 shows the relative abundances of the different groups at the phylum and class level for both years. *Deinococcus-Thermus, Proteobacteria, Chloroflexi* and *Bacteroidetes* were abundant in both samples. Comparison with a previous survey (Sánchez *et al*., 2011) indicates that most of these groups were also retrieved by different molecular methodologies. However, the contribution of each group varied depending on the technique used. The bacterial clone library over-represented the *Deinococcus-Thermus* group, while the rest of procedures showed similar results concerning this phylum. In contrast, the *Alphaproteobacteria* were over-represented by FISH (Fig. S3).

On the other hand, pyrosequencing allowed the detection of other groups that could not be recognized by other molecular techniques, such as the *Chloroflexi*, *Chlorobi*, *Deferribacteres*, *Verrumicrobia*, *Planctomycetes* and *Spirochaetes*, deepening our knowledge on the diversity of this activated sludge. Also, a certain percentage of sequences remained as unidentified bacteria (6.5% and 10.5% for years 2007 and 2008; Fig. S2). Except the *Chlorobi* and *Deferribacteres*, different pyrosequencing studies have reported the presence of these groups in conventional activated sludge samples (Sanapareddy *et al*., 2009; Kwon *et al*., 2010). However, it is remarkable that, in general, the proportions of the different groups in freshwater activated sludge were different from saline samples, and when going deeper into genus composition, the assemblage of our samples differs strongly from that previously reported. In general, prior pyrosequencing studies with different samples of activated sludge are in agreement with the predominance of the classes *Beta-* and *Gammaproteobacteria* and the phylum *Bacteroidetes* (Sanapareddy *et al*., 2009; Kwon *et al*., 2010), while in our saline activated sludge the groups that predominate are, within the phylum *Proteobacteria*, the *Alpha-* (8.0% and 7.3% in samples 2007 and 2008 respectively), *Gamma-* (19.0% and 21.4%) and *Deltaproteobacteria* (15.2% and 7.2%), as well as the *Deinococcus-Thermus* group (21.8% and 10.9%) and members of the phyla *Chloroflexi* (9.5% and 35.1%) and *Bacteroidetes* (18.3% and 2.8%). In contrast, Ye and colleagues (2002), who analysed by pyrosequencing the bacterial composition of a slightly saline activated sludge from a laboratory-scale nitrification reactor and a WWTP from Hong Kong, found that, in addition to *Proteobacteria* and *Bacteroidetes*, the phylum *Firmicutes* was also abundant in their samples; they also obtained similar groups as in the present study, such as the *Actinobacteria*, *Planctomycetes*, *Verrumicrobia*, *Deinococcus-Thermus*, *Chloroflexi* and *Spirochaetes*, although at different relative ratios, as well as different phyla not retrieved in the present work, for example the *Nitrospira*, *Chlamydiae* and TM7. Probably, differences are due to the feeding wastewater, since in our case the main influent corresponds to intermediate products of amoxicillin synthesis whereas in the other study the WWTP treated a slightly saline urban sewage from Hong Kong.

As in previous pyrosequencing studies (Keijser *et al*., 2008; Liu *et al*., 2008; Claesson *et al*., 2002), a part of sequences could only be assigned to the phylum/class level and the majority of taxa could not be classified at the genus level (74% for 2007 and 83.5% for 2008), demonstrating the extraordinary microbial diversity of activated sludge that cannot be classified using public 16S rRNA databases. Table S2 shows the taxa found in each sample in this study at the genus level, which are different from other reported genus of freshwater or either slightly saline activated sludge studies (Sanapareddy *et al*., 2009; Kwon *et al*., 2010; Ye *et al*., 2002). One of the most abundant genus in both samples is *Truepera,* a member of the phylum *Deinococcus*-*Thermus*, which includes radiation-resistant and thermophilic species. Although this phylum was also detected by pyrosequencing in a recent study with a slightly saline activated sludge (Ye *et al*., 2002), it accounted for no more than 0.6% of total community, while we found a significant percentage of sequences from this genus (21.8% and 10.9% for years 2007 and 2008 respectively).

### Diversity of nitrifying community in activated sludge

A total of 43 297 *amoA* gene sequences of good quality (11 236 reads for year 2007 and 32 061 reads for year 2008) were grouped into operational taxonomic units using uclust at 6% cut-off level. We selected a 6% cut-off to group closely related phylotypes of the *amoA* gene without losing potentially valuable information by the inclusion of phylogenetically distinct sequences. Interestingly, the diversity of the nitrifying bacterial community revealed by pyrosequencing of the *amoA* gene was very low and rarefaction analyses showed the depth of sequencing was sufficient to saturate the curve and recover the great majority. The clustering of 43 297 reads resulted in a total of only eight OTUs from which six were shared between samples as shown in the Venn diagram (Fig. 1B). The shared OTUs corresponded to 97% of total reads, which indicates that the nitrifying community was very similar both years.

**Figure 3:**
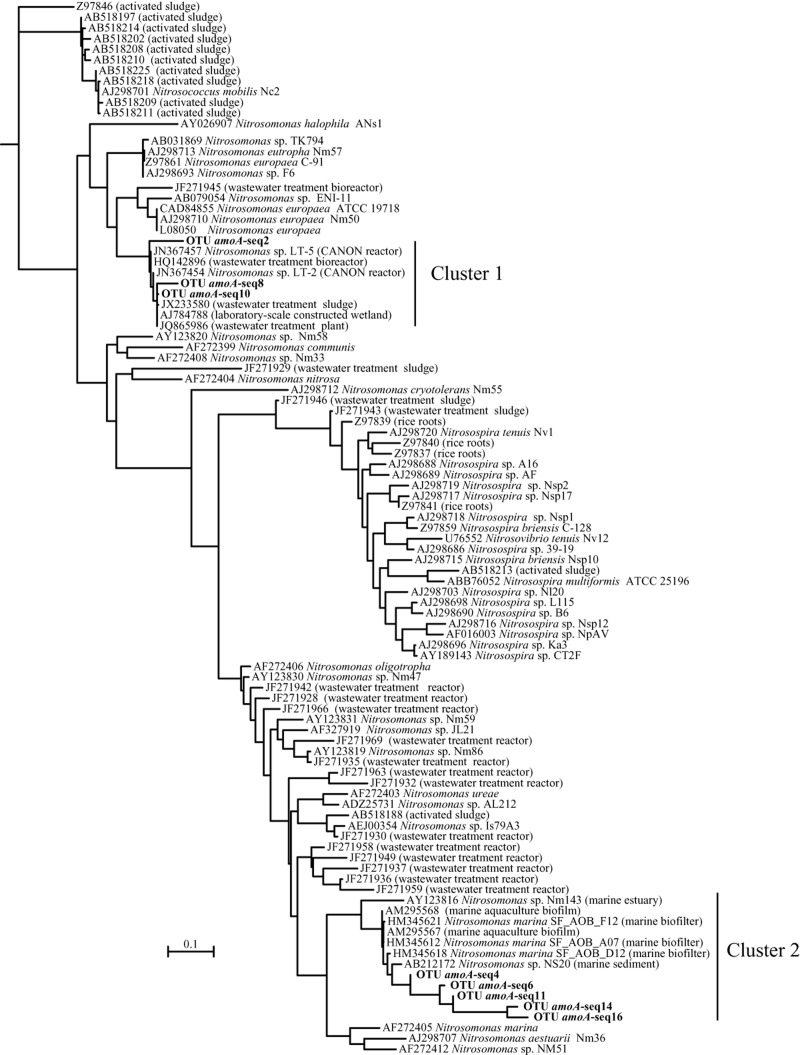
Maximum-likelihood tree of *amoA* gene. The tree was determined using approximately 491 unambiguously aligned positions of nucleic acid *amoA* sequences. Each sequence from this study (in bold) is representative of clustered *amoA* sequences in the WWTP activated sludge with an identity of 94%. Reference sequences from GenBank database are indicated by their accession number if they correspond to uncultured organisms or by the strain name if they belong to *amoA* sequences from bacterial strains. The tree was constructed with RAxML (http://bioinformatics.oxfordjournals.org/content/22/21/2688.full) using the GTRGAMMA model and an alignment made with MUSCLE (http://www.ncbi.nlm.nih.gov/pmc/articles/PMC390337/). The sequence of methane monooxygenase subunit A from *Methylococcus capsulatus* strain Bath served as outgroup (GenBank Accession No. YP_115248). The scale bar indicates substitutions per site.

All *amoA* sequences were highly related to previously described sequences in the GenBank database, both environmental and from isolates (Fig. 3). Phylogenetic analysis revealed that eight phylotypes formed two separate clusters. The first cluster, which contains three OTUs, was mostly retrieved in the 2008 library and represented 45.4% of sequences of that sample. The closest relatives in GenBank database (99% similarity) included sequences from organisms that have not been obtained from a WWTP, and *Nitrosomonas* sp. LT-2 and LT-5, isolated from a CANON reactor (98% identity). The second cluster, which contains five of the OTUs and represented the most abundant phylotypes in both samples, was most closely related (94% identity) to cultured representatives of strains of *Nitrosomonas marina* isolated from a biofilter of a recirculating shrimp aquaculture system (GenBank Accession No. HM345621, HM345612 and HM345618) and *Nitrosomonas* sp. NS20 isolated from coastal marine sediments. This cluster virtually represented all sequences (99.99%) in the sample of 2007 whereas in 2008 it comprised 54.6%.

These data are consistent with previous results found by Ye and colleagues (2002) in slightly saline activated sludge, which showed that *Nitrosomonas*, together with *Nitrospira*, was the dominant nitrifying genera, and also with the study by Park and colleagues (2009), who identified that a specific ammonia-oxidizing bacteria belonging to the *Nitrosomonas europaea* lineage was dominant in a full-scale bioreactor treating saline wastewater due to its adaptation to high-salt conditions. In general, nitrosomonads are also responsible for ammonia oxidation in conventional WWTPs (Purkhold *et al*., 2000; Zhang *et al*., 2011b). However, we did not retrieve *Nitrosomonas* in the pyrosequencing 16S rRNA libraries or previously in DGGE gels, clone libraries and FISH (Sánchez *et al*., 2011) probably due to their low abundance. Thus, pyrosequencing of functional genes such as *amoA* revealed the presence of particular groups which could not be retrieved when analysing the 16S rRNA, demonstrating its value to deepen into the functionality of microbial populations when targeting specific genes. The only nitrifier that could be retrieved in our samples by 16S rRNA pyrosequencing was *Nitrosococcus*, a *Gammaproteobacteria* which just represented 0.3% and 0.5% of the total reads for years 2007 and 2008 respectively, and has also been reported to be an important nitrifier in some activated sludges (Juretschko *et al*., 1998; Raszka *et al*., 2011). Thus, since it was actually detectable in the general bacterial 16S rRNA gene population, it could also participate in ammonia oxidization together with the *Betaproteobacteria,* despite previous efforts for amplifying the gammaproteobacterial *amoA* gene yielded negative results (Sánchez *et al*., 2011).

On the other hand, we know that nitrification and denitrification are central processes in our system, since a nitrification fraction of 98% and a total nitrogen removal over 80% have been reported (M.I. Maldonado, pers. comm.). In fact, Yu and Zhang (2012), when applying both metagenomic and metatranscriptomic approaches to characterize microbial structure and gene expression of an activated sludge community from a municipal WWTP in Hong Kong found that nitrifiers such as *Nitrosomonas* and *Nitrospira* had a high transcription activity despite presenting a very low abundance (they accounted only 0.11% and 0.02% respectively in their DNA data set), and the results from Zhang and colleagues (2011b) indicated that the abundance of ammonia-oxidizing bacteria in the activated sludge from different WWTPs was very low. Similarly, our results suggest that *Nitrosomonas* could be responsible of nitrification although showing a low abundance. Also, it may be possible that other genera different from the well-known *Betaproteobacteria* could contribute to nitrification activity.

In fact, different studies have reported the autotrophic oxidation of ammonia by members of the domain *Archaea*. The crenarchaeon *Nitrosopumilus maritimus* is able to oxidize ammonia to nitrite under mesophilic conditions (Könneke *et al*., 2005), while ammonia-oxidizing *Archaea* occurred in activated sludge bioreactors used to remove ammonia from wastewater (Park *et al*., 2006). However, amplification of the *amoA* gene to detect the presence of archaeal nitrifiers yielded negative results in our samples. In fact, *Archaea* accounted only for 6% of DAPI counts, and all sequences retrieved previously in an archaeal clone library were related to methanogenic archaea (Sánchez *et al*., 2011). Other studies have also demonstrated the presence of methanogens in aerated activated sludge but, although active, they played a minor role in carbon and nitrogen turnover (Gray *et al*., 2002; Fredriksson *et al*., 2012).

Interestingly, different heterotrophic bacteria, such as *Bacillus* sp. (Kim *et al*., 2005), *Alcaligenes faecalis* (Liu *et al*., 2012), *Marinobacter* sp. (Hai-Yan *et al*., 2002), *Achromobacter xylosoxidans* (Kundu *et al*., 2012) and *Pseudomonas* sp. (Su *et al*., 2006) have been described as potential nitrifiers, and remarkably, some of these genera have been isolated from our activated sludge by culture-dependent techniques (data not shown); for instance, some strains were identified as *Bacillus* sp., *Alcaligenes* sp., *Marinobacter hydrocarbonoclasticus* and *Pseudomonas* sp.

In contrast, sequences of *Nitratireductor* sp., a denitrifying microorganism, have been retrieved with different molecular methods (pyrosequencing in this study and DGGE and clone library in Sánchez *et al*., 2011), while other sequences from potential denitrifiers have been recovered only by 454-pyrosequencing, such as *Leucobacter* sp., *Caldithrix* sp*.*, *Castellaniella* sp. and *Halomonas* sp. Besides, other candidates for denitrifying bacteria have been isolated by culture-dependent techniques, such as *Alcaligenes* sp.*, Bacillus* sp.*, Paracoccus* sp.*, Pseudomonas* sp. and *Marinobacter* sp. (data not shown). Other genera retrieved by pyrosequencing were related to the nitrogen fixation process, that is, *Microbacterium* sp., *Aminobacter* sp. and *Spirochaeta* sp., while *Sphingomonas* was detected by clone library and culture-dependent methods.

Summarizing, we can conclude that the bacterial diversity in the activated sludge of the seawater-processing plant was high as previously observed in conventional WWTPs. However, the composition of the bacterial community differed strongly from other plants, and was dominated by *Deinococcus-Thermus, Proteobacteria, Chloroflexi* and *Bacteroidetes*. Previous analyses by clone library, DGGE and FISH were not enough to reflect the profile of the bacterial community in wastewater sludge and although pyrosequencing was a powerful tool to define the microbial composition deeper sequencing is required. Despite nitrification rates were high in the system, known ammonia-oxidizing bacteria were not identified by means of 16S rRNA studies and analysis of the specific functional gene *amoA* was required to reveal the presence and identity of the bacteria responsible for this process. These results suggest that only a few populations of low abundant but specialized bacteria likely with high transcription activity are responsible for removal of ammonia in these systems. However, further studies to isolate the key microorganisms involved in ammonia-oxidation will be essential in order to understand this process in saline WWTPs.

## Supporting information

Table S1 Summary of clean sequences, singletons and operational taxonomic units (OTUs) in the two data sets analysed.

Table S2 Genera identified from pyrosequencing and cloning in the saline activated sludge.

Fig S1 Rank abundance curves of OTUs defined by a 3% sequence variation for 16S rRNA (A) and 6% for amoA (B) genes in the activated sludge.

Fig S2 Taxonomic composition by phylum and class for the sequences retrieved in 2 consecutive years.

Fig S3 Percentage of relative intensity of DGGE bands, clones (library), probe positive cells scaled to Eub probes (FISH) and reads (454) affiliated to different phylogenetic groups from samples D07 and D08. Data from DGGE, cloning and FISH have been extracted from Sánchez and colleagues (2011). [*Alphaproteobacteria* (Alpha), *Betaproteobacteria* (Beta), *Gammaproteobacteria* (Gam), *Deltaproteobacteria* (Delta), *Bacteroidetes* (Bact), *Firmicutes* (Firm), *Deinococcus-Thermus* (DT), *Actinobacteria* (Actino), *Chloroflexi*(Chloroflexi), *Def* (Deferribacteres), *Chlorobi* (Chlorobi), Not determined (Not det).]
