# Supplementary material for: Assessing bacterial diversity in a seawater-processing wastewater treatment plant by 454-pyrosequencing of the 16S rRNA and amoA genes
Source: Microb Biotechnol. 2013 Apr 10;6(4):435–42. doi: 10.1111/1751-7915.12052 (PMC3917478; doi:10.1111/1751-7915.12052)
Supplement: Table S1 — Summary of clean sequences, singletons and operational taxonomic units (OTUs) in the two data sets analysed. [file mbt20006-0435-sd1.docx]

*Sampling and DNA extraction*

Samples of aerated mixed activated sludge from a seawater processing wastewater treatment plant located in Almeria (southeast Spain) were collected in December 2007 and November 2008 in a 1 L sterile bottle and stored at 4ºC. A 50 ml subsample was centrifuged and the pellet was stored at –20ºC until processing. Upon thawing, the total community DNA was extracted using the DNA Power Soil kit from MOBIO (12888-50) following manufacture’s instructions. DNA was quantified spectrophotometrically with a SmartSpec Plus (Biorad) and a subsample was sent to pyrosequencing.

*Sequence generation and processing*

Pyrosequencing of 16S rRNA and *amoA* genes was performed by the Research and Testing Laboratory (Lubbock, TX, USA; http://www.medicalbiofilm.org) by using the bTEFAP method as described previously (Dowd et al., 2008). For 16S rRNA gene eubacterial primers 28F (5’-GAGTTTGATCNTGGCTCAG-3’) and 519R (5’-GTNTTACNGCGGCKGCTG-3’) were used whereas for *amoA* amplification primer set used was amoA-1F (5’-GGGGTTTCTACTGGTGGT-3’) and amoA-2R (5’-CCCCTCKGSAAAGCCTTCTTC-3’) (Rotthauwe et al., 1997). This resulted in amplicon fragments of 492 and 491 bp long respectively suitable for the 454 GL FLX technology.

Pyrosequencing data generated was processed using the QIIME pipeline (Quantitative Insights Into Microbial Ecology, Caporaso et al., 2010). QIIME takes all sequences from a pyrosequencing run and assigns sample IDs using a mapping file and the barcode assigned to each sample. After sample IDs were assigned, a step of sequence filtration was performed before denoising. Both for 16S rRNA gene and *amoA* gene, sequences were removed from the subsequent analyses if they were shorter than 150bp or longer than 600bp, had an average quality score <25 calculated in sliding windows of 50bp, contained more than two ambiguous characters or had an uncorrectable barcode. The remaining sequences were run through Denoiser to reduce the impact of pyrosequencing errors (Reeder and Knight, 2010). Clean sequences were then grouped into operational taxonomic units (OTUs) or phylotypes using uclust (Edgar, 2010) with a minimum identity of 97% for 16S rRNA and 94% for *amoA* gene sequences. A representative sequence from each phylotype was chosen by selecting the most abundant sequence within that particular phylotype and was used for taxonomic identification. The resulting 16S rRNA gene representative sequences were checked for chimeras using ChimeraSlayer (Haas et al., 2011) in mothur (Schloss et al., 2009). For *amoA* gene, due to the low number of OTUs and the lack of public *amoA* databases, the presence of chimeras was checked manually using BLAST against NCBI’s non-redundant database (http://www.ncbi.nlm.nih. gov). The identify of 16S rRNA phylotypes was determined using the RDP Classifier (Wang et al., 2007) in QIIME, whereas *amoA* gene phylotypes were identified using BLAST against NCBI’s non-redundant database (http://www.ncbi.nlm.nih. gov). Chao1 diversity metrics and rarefactions curves were computed in QIIME. A summary of sequence information regarding both datasets can be found in Table S1. Sequence data has been deposited in the MG-RAST public database (<http://metagenomics.anl.gov/>) under ID numbers 4509194.3 (16S rRNA) and 4509195.3 (*amoA*).

**References**

Caporaso, J.G., Kuczynski, J., Stombaugh, J., Bittinger, K., Bushman, F.D., Costello, E.K., Fierer, N., González Pena, A., et al. (2010) Qiime allows analysis of high-throughput community sequencing data. *Nature Methods* **7:** 335-336.

Dowd, S.E., Sun, Y., Wolcott, R-D., Domingo, A., Carroll, J.A. (2008) Bacterial tag-encoded FLX amplicon pyrosequencing (bTEFAP) for microbiome studies: bacterial diversity in the ileum of newly weaned *Salmonella*-infected pigs. *Foodborne Pathog Dis* **5:** 459-472.

Edgar, R.C. (2010) Search and clustering orders of magnitude faster than BLAST, *Bioinformatics* **26:** 2460-2461.

Haas, B.J., Gevers, D., Earl, A.M., Feldgarden, M., Ward, D.V., Giannoukos, G. *et al*. (2011). Chimeric 16S rRNA sequence formation and detection in Sanger and 454-pyrosequenced PCR amplicons. *Genome Res* **21:** 494–504.

Reeder, J., and Knight, R. (2010). Rapidly denoising pyrosequencing amplicon reads by exploiting rank-abundance distributions. *Nat Methods* **7:** 668–669.

Rotthawe, J.H., Witzel, K.P., and Liesack, W. (1997) The ammonia monooxigenase structural gene amoA as a functional marker: molecular fine-scale analysis of natural ammonia-oxidizing populations. *Appl Environ Microbiol* **63:** 4704-4712.

Schloss, P.D., Westcott, S.L., Ryabin, T., Hall, J.R., Hartmann, M., Hollister, E.B. *et al*. (2009). Introducing mothur: open-source, platform-independent, community-supported software for describing and comparing microbial communities. *Appl Environ Microbiol* **75:** 7537–7541.

Wang, Q., Garrity, G.M., Tiedje, J.M., and Cole, J.R. (2007) Naive Bayesian classifier for rapid assignment of rRNA sequences into the new bacterial taxonomy. *Appl Environ Micr*obiol **73:** 5261-5267.
