# Supplementary material for: Assessing bacterial diversity in a seawater-processing wastewater treatment plant by 454-pyrosequencing of the 16S rRNA and amoA genes
Source: Microb Biotechnol. 2013 Apr 10;6(4):435–42. doi: 10.1111/1751-7915.12052 (PMC3917478; doi:10.1111/1751-7915.12052)
Supplement: Table S2 — Genera identified from pyrosequencing and cloning in the saline activated sludge. [file mbt20006-0435-sd2.docx]

|  | **Clean tags** | **Singletons** | **OTUs** | **Unique OTUs** |
| --- | --- | --- | --- | --- |
| *16S rRNA gene* | 16176 | 136 | 320 | - |
| D07 | 8010 | 72 | 201 | 94 |
| D08 | 8166 | 64 | 226 | 119 |
|  |  |  |  |  |
| *amoA gene* | 43297 | 0 | 8 | - |
| D07 | 11236 | 0 | 6 | 0 |
| D08 | 32061 | 0 | 8 | 0 |
