# Supplementary material for: Assessing bacterial diversity in a seawater-processing wastewater treatment plant by 454-pyrosequencing of the 16S rRNA and amoA genes
Source: Microb Biotechnol. 2013 Apr 10;6(4):435–42. doi: 10.1111/1751-7915.12052 (PMC3917478; doi:10.1111/1751-7915.12052)
Supplement: Fig S1 — Rank abundance curves of OTUs defined by a 3% sequence variation for 16S rRNA (A) and 6% for amoA (B) genes in the activated sludge. [file mbt20006-0435-sd3.docx]

**Percentage of sequences**

**______________________________________________**

**Phylum Genus DER07 DER07 DER08**

**(pyrosequencing) (cloning) (pyrosequencing)**

Actinobacteria *Leucobacter* 0.6 0 0.8

Actinobacteria *Microbacterium* 0.4 0 0.7

Bacteroidetes *Gracilimonas* 0.03 0 0.1

Bacteroidetes *Cryomorpha* 1.7 0 0

Bacteroidetes *Aequorivita* 0.05 0 0.01

Chloroflexi *Caldilinea* 0.1 0 0.24

Deferribacteres *Caldithrix* 0.3 0 0.5

Deinococcus-Thermus *Truepera* 21.8 77.4 10.9

Alphaproteobacteria *Hyphomonas* 0.04 0 0

Alphaproteobacteria *Aminobacter* 0.01 0 0.05

Alphaproteobacteria *Nitratireductor* 0.02 1.8 0.15

Alphaproteobacteria *Porphyrobacter* 0.01 0 0

Betaproteobacteria *Castellaniella* 0.01 0 0

Betaproteobacteria *Schlegelella* 0.01 0 0

Gammaproteobacteria *Aeromonas* 0.01 0 0

Gammaproteobacteria *Haliea* 0.06 0 0.05

Gammaproteobacteria *Nitrosococcus* 0.3 0 0.5

Gammaproteobacteria *Methylohalomonas* 0.4 4 2.3

Gammaproteobacteria *Halomonas* 0.02 0 0.09

Gammaproteobacteria *Francisella* 0.15 0 0.08

Gammaproteobacteria *Methylophaga* 0 0 0.02

Spirochaetes *Spirochaeta* 0 0 0.01
