# Supplementary figures and images for: Assessing bacterial diversity in a seawater-processing wastewater treatment plant by 454-pyrosequencing of the 16S rRNA and amoA genes
Source: Microb Biotechnol. 2013 Apr 10;6(4):435–42. doi: 10.1111/1751-7915.12052 (PMC3917478; doi:10.1111/1751-7915.12052)

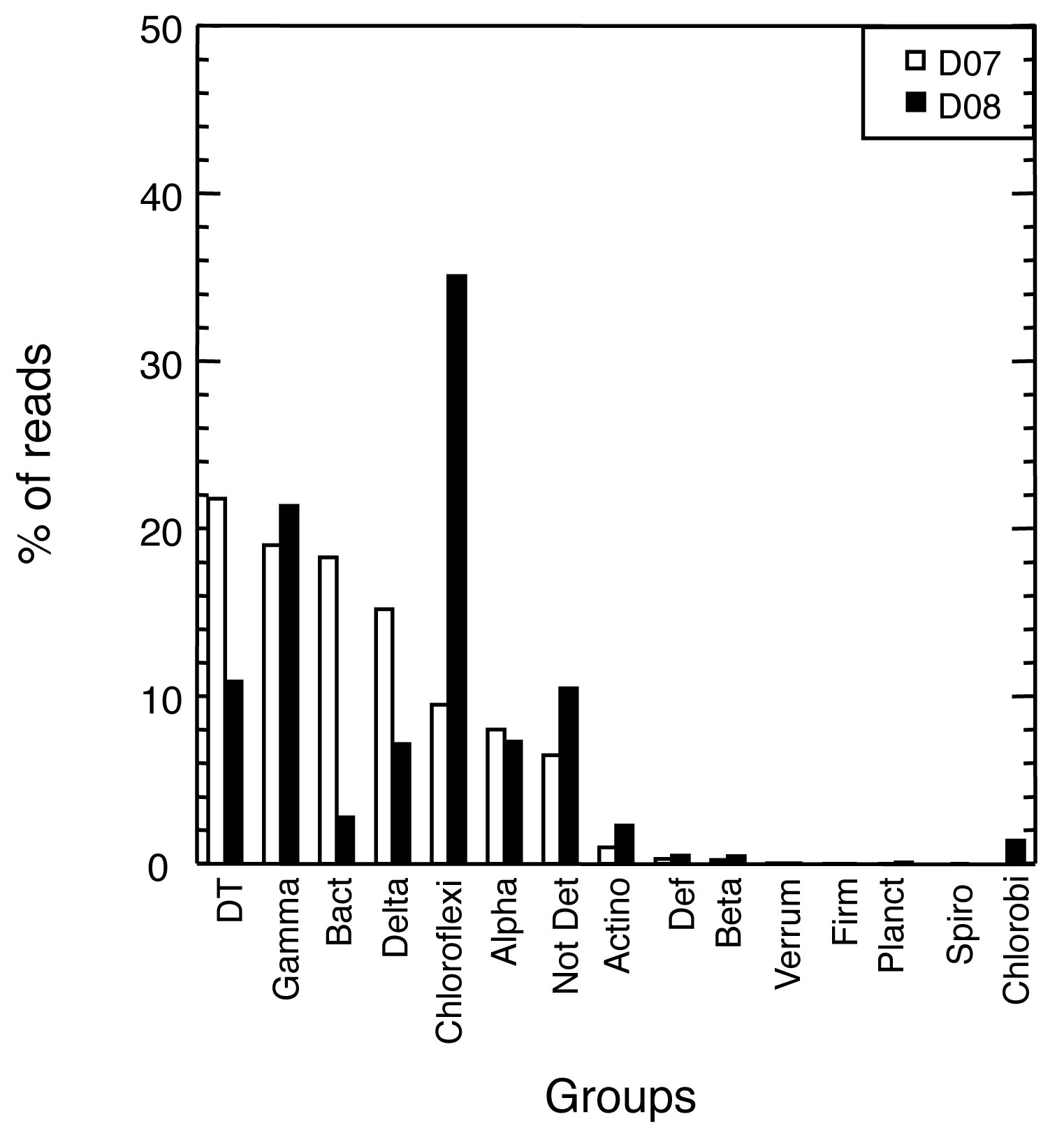

Supplement: Fig S3 — Percentage of relative intensity of DGGE bands, clones (library), probe positive cells scaled to Eub probes (FISH) and reads (454) affiliated to different phylogenetic groups from samples D07 and D08. Data from DGGE, cloning and FISH have been extracted from Sánchez and colleagues (2011). [Alphaproteobacteria (Alpha), Betaproteobacteria (Beta), Gammaproteobacteria (Gam), Deltaproteobacteria (Delta), Bacteroidetes (Bact), Firmicutes (Firm), Deinococcus-Thermus (DT), Actinobacteria (Actino), Chloroflexi(Chloroflexi), Def (Deferribacteres), Chlorobi (Chlorobi), Not determined (Not det).] [file mbt20006-0435-sd5.tif]

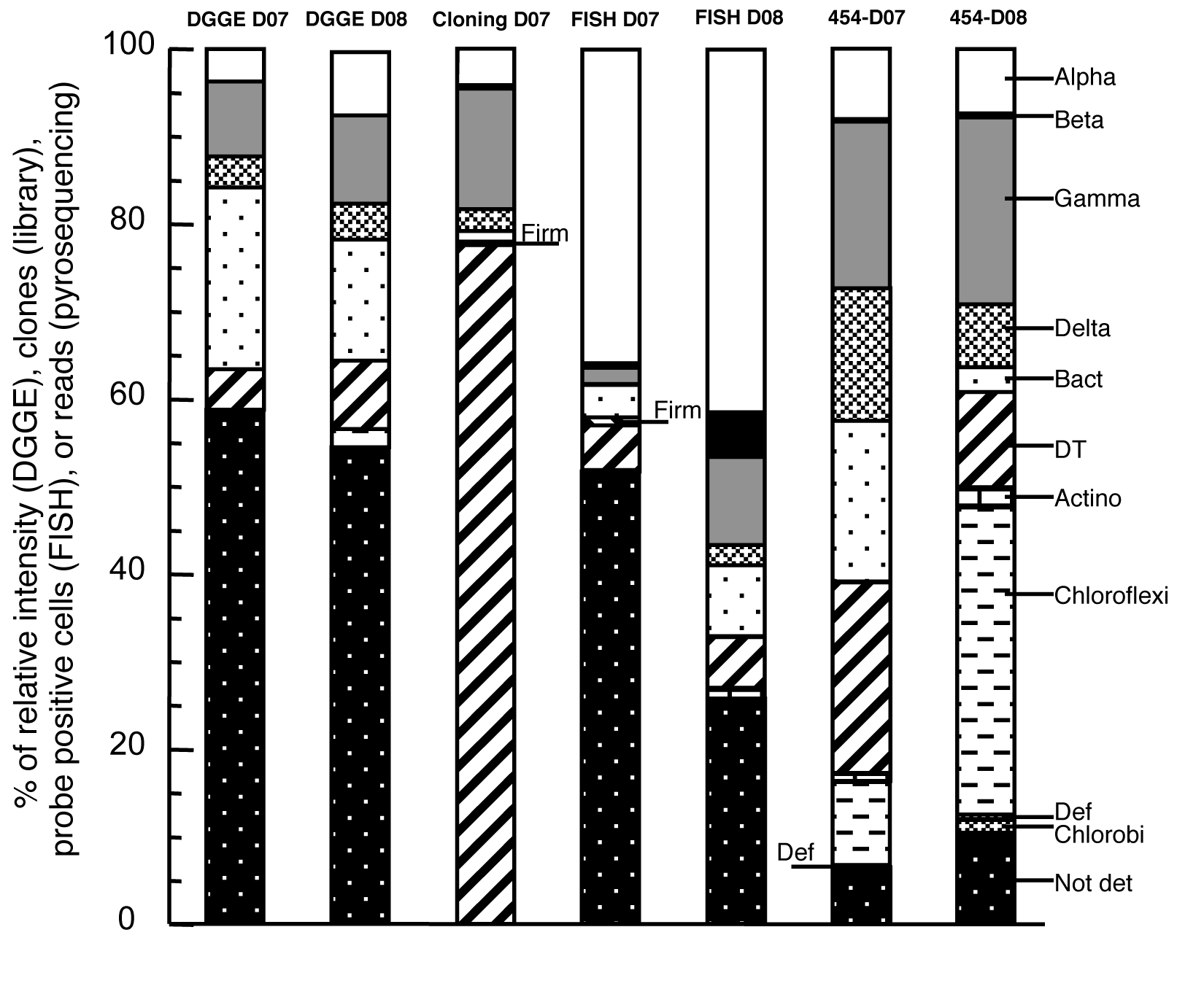

Supplement: Supplementary file 6 [file mbt20006-0435-sd6.tif]
